# Supplementary material for: The interactome of intact mitochondria by cross-linking mass spectrometry provides evidence for coexisting respiratory supercomplexes
Source: Mol Cell Proteomics. 2017 Dec 8;17(2):216–32. doi: 10.1074/mcp.RA117.000470 (PMC5795388; doi:10.1074/mcp.RA117.000470)
Supplement: Supplemental Data [file supp_RA117.000470_133922_0_supp_23978_fzffwf.zip › spectra_annotation/mito_DR_spectra_annotation/497-1-19-1-5-1.pdf]

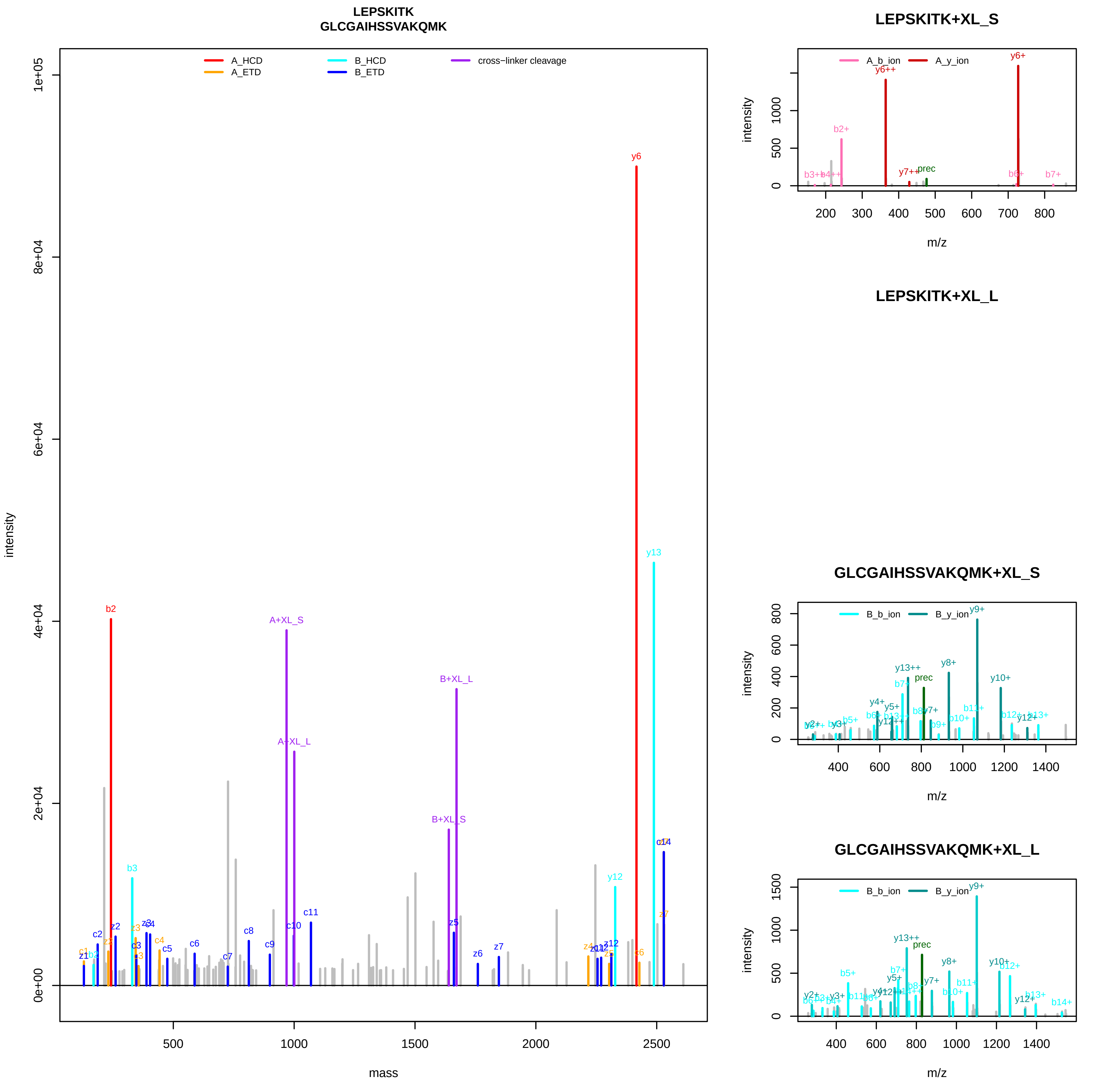
LEPSKITK
GLCGAIHSSVAKQMK
A_HCD
A_ETD
B_HCD
B_ETD
cross−linker cleavage
intensity
mass
0e+00
2e+04
4e+04
6e+04
8e+04
1e+05
500
1000
1500
2000
2500
b2
y6
y13
A+XL_S
A+XL_L
B+XL_S
B+XL_L
b3
y12
c14
c11
c10
c9
c8
c7
c6
c5
c4
c3
c2
z1
z2
z3
z5
z6
z7
z12
LEPSKITK+XL_S
A_b_ion
A_y_ion
y6+
y6++
b2+
y7++
prec
b3++
b4++
b6+
b7+
m/z
LEPSKITK+XL_L
GLCGAIHSSVAKQMK+XL_S
B_b_ion
B_y_ion
y9+
y8+
y13++
y10+
b7+
b11+
b12+
b13+
y12+
GLCGAIHSSVAKQMK+XL_L
b14+
